# Application of Shear Horizontal Surface Acoustic Wave (SH-SAW) Immunosensor in Point-of-Care Diagnosis

**DOI:** 10.3390/bios13060605

**Published:** 2023-06-01

**Authors:** Chia-Hsuan Cheng, Hiromi Yatsuda, Mikihiro Goto, Jun Kondoh, Szu-Heng Liu, Robert Y. L. Wang

**Affiliations:** 1 Graduate School of Science and Technology, Shizuoka University, 3-5-1 Johoku, Naka-ku, Hamamatsu-shi 432-8561, Japan; joshcheng@tst.bio (C.-H.C.); yatsuda.hiromi@tst.bio (H.Y.); kondoh.jun@shizuoka.ac.jp (J.K.); 2 tst Biomedical Electronics Co., Ltd., Taoyuan 324403, Taiwan; justinliu@tst.bio; 3 Japan Radio Co., Ltd., Nagano 381-2289, Japan; goto.mikihiro@jrc.co.jp; 4 Biotechnology Industry Ph.D. Program, Chang Gung University, Taoyuan 33302, Taiwan; 5 Kidney Research Center and Department of Nephrology, Chang Gung Memorial Hospital, Linkou 33305, Taiwan; 6 Department of Biomedical Sciences, College of Medicine, Chang Gung University, Taoyuan 33302, Taiwan

**Keywords:** surface acoustic wave, SAW, SH-SAW, point-of-care testing, POCT, whole blood measurement, simulation, C-reactive protein, lipoprotein, apolipoprotein

## Abstract

Point-of-care testing (POCT), also known as on-site or near-patient testing, has been exploding in the last 20 years. A favorable POCT device requires minimal sample handling (e.g., finger-prick samples, but plasma for analysis), minimal sample volume (e.g., one drop of blood), and very fast results. Shear horizontal surface acoustic wave (SH-SAW) biosensors have attracted a lot of attention as one of the effective solutions to complete whole blood measurements in less than 3 min, while providing a low-cost and small-sized device. This review provides an overview of the SH-SAW biosensor system that has been successfully commercialized for medical use. Three unique features of the system are a disposable test cartridge with an SH-SAW sensor chip, a mass-produced bio-coating, and a palm-sized reader. This paper first discusses the characteristics and performance of the SH-SAW sensor system. Subsequently, the method of cross-linking biomaterials and the analysis of SH-SAW real-time signals are investigated, and the detection range and detection limit are presented.

## 1. Introduction

An acoustic wave biosensor is a unique device that uses mechanical vibrations with frequencies ranging from a few megahertz (MHz) to several gigahertz (GHz) to detect binding events on the surface of a chip. These biosensors have some advantages, including being label-free, small in size, reasonable in price, and capable of producing quantitative results using an electrical circuit. Many studies have been published; these have addressed thickness shear mode (TSM) [1,2,3], acoustic plate mode (APM) [4], surface acoustic wave (SAW) [5,6,7], bulk acoustic wave (BAW) [8], and shear horizontal surface acoustic wave (SH-SAW) [5,9,10,11,12]. There are many varieties of different acoustic modes, different frequencies, and different substrate materials. However, only a few products on the market are in use: TSM, BAW, and SH-SAW. Quartz crystal microbalance (QCM) has been widely used for a long time in a variety of different applications [13,14,15]. One of the successful commercial applications of QCM is an odor sensor [16,17]. Multiple QCM sensors have the property of responding to different odors in the air, generally using machine learning with pattern recognition systems [18,19]. In addition, many researchers have used QCM systems to study binding events in biological applications [20]. A BAW biosensor system consisting of a desktop reader and a microfluidic cartridge has been introduced for medical applications [21]. Qorvo Biotechnologies has obtained an emergency use authorization (EUA) from the FDA for the rapid detection of the COVID-19 antigen by using BAW at the point of care [22]. On the other hand, SH-SAW sensors are popular in liquid phase applications [23,24,25,26,27,28,29]. In addition, a unique SH-SAW biosensor system has been commercialized for medical applications. The system consists of a palm-sized reader and a disposable cartridge with a simple structure like a lateral flow test cartridge.

This paper outlines an SH-SAW biosensor system that has been successfully commercialized for medical use. The system has three main unique features: a disposable test cartridge with an SH-SAW sensor chip, a mass-produced bio-coating, and a palm-sized reader. The sensor chip has a unique air chamber structure that protects the electrical transducers from liquids and has a sensing area exposed to apply a drop of a sample. The sensing area of the chip has two channels: one for sensing and one for reference. These two channels have dried proteins on the gold surface, which are applied in the factory. To apply the proteins to the gold surface of the chip, several machines were designed and a pilot line of more than 10 machines was set up in series for automatic coating. This pilot line has a capacity of several million chips per year. The palm-sized reader measures the phase and amplitude changes of the SH-SAW in real time and estimates the concentration using a calibration curve, which is provided by a QR code for each test cartridge. The reader and sensor chip are common platforms for different markers. This paper demonstrates the performance of a number of different markers measured using this system, including C-reactive protein (CRP), lipoprotein (a) (Lp(a)), apolipoprotein B (ApoB) [30], and anti-SARS-CoV-2 Spike protein antibody [12].

## 2. Shear Horizontal Surface Acoustic Wave (SH-SAW) Sensor System

### 2.1. SH-SAW Sensor Chip

A 250 MHz two-channel SH-SAW delay line biosensor was designed and evaluated on a 36° Y-cut 90° X-propagated quartz with a thickness of 0.5 mm [31]. The quartz substrate was chosen because it has a smaller temperature coefficient than LiTaO_3_ or LiNbO_3_ substrates. For commercialization, we modified the previously designed delay line [31]. To reduce the size of chip, we designed a reflective delay line. The floating electrode unidirectional transducers (FEUDTs) [32], reflector, and sensing areas form a gold film with a thickness of about 117.5 nm on the chip surface. The FEUDTs have an aperture of about 0.4 mm and 80 finger pairs. A 250 MHz SH-SAW with a wavelength of about 20 μm is excited at the FEUDT; propagates to the reflector, where it is reflected; and then propagates back to the FEUDT, where it is received. The size of each sensing zone is approximately 0.56 mm × 1.8 mm. To protect the transducers, a unique microfabricated air cavity structure was developed on top of the FEUDTs. The air cavity consists of epoxy resin walls surrounding the FEUDTs and a glass coverlid [31]. This structure minimizes the attenuation of SH-SAWs, and liquid reagents and samples can be applied directly to the chip surface. We have tested SH-SAW biosensors at different frequencies, from 50 MHz to 500 MHz. Currently, we have chosen an SH-SAW delay line chip with a time delay of about 1 microsecond and an operating frequency of 250 MHz because its signal-to-noise ratio is critical for the sensor system.

**Figure 1:**
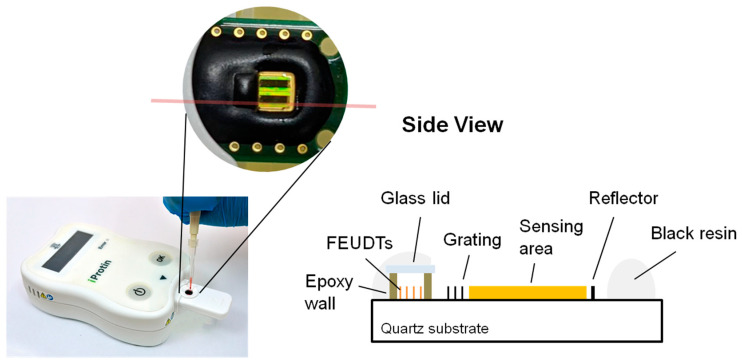
SH-SAW biosensor chip with POCT platform.

The two-channel SH-SAW delay line biosensor chip has a size of approximately 3 mm × 6 mm; one is the reference channel and the other is the capture channel. The chip is mounted on a printed circuit board (PCB) with dimensions of about 10 mm × 23 mm, and the signal pads and ground pads are connected by bonding wires. After that, the bonding wires and some areas of the chip are molded with epoxy resin. As shown in Figure 1, there is an open area of about 3 mm square around the black epoxy resin, which facilitates the retention of the sample liquid. The two black rectangles within the open area are a reference channel and a capture channel with a gold film.

### 2.2. Reader (to Measure Phase and Amplitude Changes of SH-SAWs)

In SH-SAW sensor systems, some binding events on the chip surface cause velocity and amplitude changes in the SH-SAWs, which are caused by mass and viscosity changes [31,33]. There are usually two ways to understand the velocity changes: measuring the resonant frequency changes and measuring the electrical phase changes. In the case of frequency measurement systems, this system can provide higher performance because we can obtain a very high frequency resolution using a very high Q SAW-based resonator. However, in the case of liquid phase applications, the liquid sample should significantly reduce the Q factor of the sensor device, and the electrical properties of the sample may also affect the resonant frequency.

**Figure 2:**
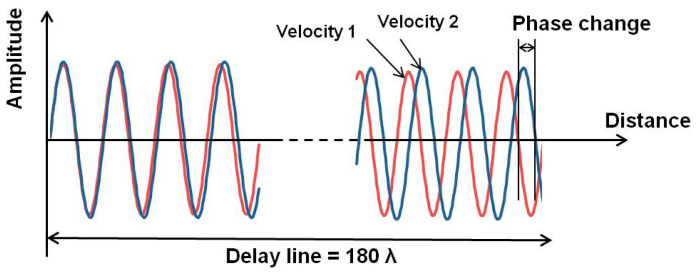
Phase change due to SH-SAW velocity change.

We developed an electrical phase measurement system for the delay line that has a sensor area separated from the electrical transducers. Thus, we can observe the binding events of biological materials under electrically stable conditions. The electrical phase and amplitude of the SH-SAW propagating over the sensing area can be measured from the difference between the input and output signals of the transducer. We have developed a custom integrated circuit chip to measure electrical phases and amplitudes [34]. The change in the velocity of the SH-SAW leads to a change in the wavelength of the SH-SAW, which in turn leads to a change in the phase of the electrical signal propagating over the sensing area of the SH-SAW. As shown in Figure 2, the larger the sensing length, the larger the electrical phase change due to the velocity change of the SH-SAW. The chip has been designed for a maximum of four-channel measurements for future applications. A palm-sized reader has been implemented with a custom IC chip that measures the phase and amplitude changes of the SH-SAWs and calculates the concentration of the sample using a four-parameter logistics (4PL) curve, which is obtained from a quick response (QR) code.

This reader is a simple electronic device with low power consumption and can be operated with four AAA batteries. The reader does not require any complex mechanical systems such as a microfluidic system, as no washing system is required. The current reader weighs about 250 g and includes a QR code reader module, near field communication (NFC) module, display module, and batteries. This is easy for physicians to carry to the patient’s home. On the other hand, if we use a smart phone to assist the reader, the reader does not need a QR code reader module, NFC module, display module, and battery. In this case, the size and cost of the reader will be significantly reduced, and it may be suitable for home use.

## 3. Basic Performance of the SH-SAW Sensor Chip

### 3.1. Simulation Model for the Evaluation of SH-SAW Performance

**Figure 3:**
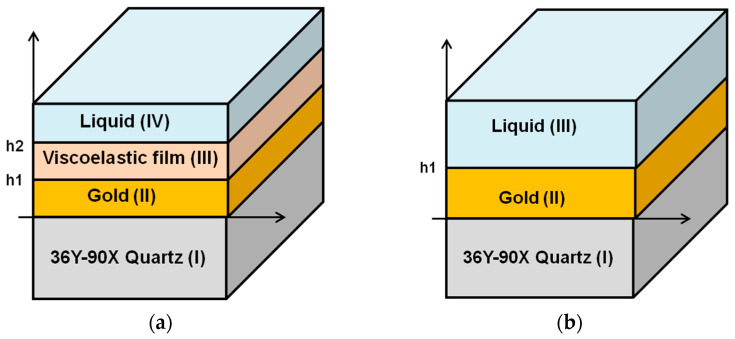
The model structures of simulation program. (**a**) the substrate layer (I) is 36Y-90X quartz, the metal layer (II) is gold, the additional material layer (III) is the protein layer and the liquid layer (IV) is water or a buffer; (**b**) The substrate layer (I) is 36Y-90X quartz, the metal layer (II) is gold and the liquid layer (III) is a glycerol-water mixture.

To understand the performance of the SH-SAW sensors, numerical computational analysis of the SH-SAW has been investigated [31,33,35]. The model structures are shown in Figure 3a,b. There are multiple layers: substrate (I)/metal(II)/liquid(III) or substrate (I)/metal(II)/viscoelastic film(III)/liquid(IV). In Figure 3a, the substrate layer (I) is 36Y-90X quartz, the metal layer (II) is gold, the additional material layer (III) is a protein layer, and the liquid layer (IV) is water or a buffer [31,33]. In this review, a three-layer model as shown in Figure 3b was used to calculate the glycerol-water mixture. The substrate layer (I) is 36Y-90X quartz, the metal layer (II) is gold, and the liquid layer (III) is a glycerol-water mixture [35,36]. In these calculations, we used an improved numerical computational method for these model structures [31,34,36]. This method was modified from two computational methods: one is based on Campbell and Jones [37], and the other is based on Moriizumi, Unno and Shiokawa [38]. This calculation method has been reported in detail [39]. In the calculation of glycerol-water mixtures, the density and viscosity of the liquid were used for layer (III). Since the solution was approximated as an uncompressed isotopic medium, the shear modulus of elasticity can be defined by the viscosity [39]. The material constants for different concentrations of glycerol mixtures were obtained from the technical data handbook [40]. We used the following values in our calculations, as shown in Table 1. This numerical calculation method [31,34,36] is very adequate for SH-SAW sensor devices on quartz. On the other hand, another analytical model for SH-SAW devices using FEM (Finite Element Method) will be investigated in the near future.

### 3.2. Mass Loading Sensitivity in Air

The mass sensitivity of this SH-SAW delay line sensor is evaluated by the center frequency changes for different gold film thicknesses. In our experiments, the center frequency variation is 440 ppm at 250 MHz SH-SAW with a gold film thickness change of 1 nm, i.e., 0.11 MHz. As shown in Table 2, the SH-SAW velocities (*V_m_*) on the 117.5 nm and 116.5 nm gold films were 4754.52 m/s and 4759.59 m/s, respectively. According to our calculation, the SH-SAW velocity (*V_o_*) on the free surface is 5089.31 m/s. If we assume a metallization ratio is 0.5, a *V_m_* of 4754.52, and a *V_o_* of 5089.31, we can obtain a center frequency change of 551 ppm and a gold film thickness change of 1 nm for the 250 MHz SH-SAW sensors.

On the other hand, the calculated SH-SAW velocity change on the gold film in the air between 117.5 nm and 116.5 nm thickness is 1066 ppm. This means that the mass loading sensitivity of the SH-SAW delay line sensor is 1066 ppm per 1 nm gold film thickness change in the air. Since the sensing length of this delay line sensor chip is 3.6 mm, a 1 nm change in gold film thickness produces a phase shift of 72.6 degrees in the air. If the minimum phase resolution of this system is 0.1 degrees, then the minimum resolution of the gold film thickness change would be 0.0138 nm in the air.

Compared with QCM, the frequency change of QCM was calculated by the equation:

(1) ∆f=−2f02(μqρq)12∆m,

where ∆*f* Hz is the measured frequency shift, $f_{0}$ Hz the resonant frequency of the quartz crystal, and ∆m kg·m^−^^2^ the mass change per unit area at the QCM surface. $\mu_{q}=$2.947 × 10^10^ kg·m^−^^1^·s^−^^2^ the shear modulus of quartz, and $\rho_{q}=$ 2.648 × 10^3^ kg·m^−^^3^ the density of quartz. The mass sensitivity of QCM is 0.606 ng/cm^2^ per 1 Hz at 27 MHz. The frequency change of this QCM for a 1 nm gold film thickness surface change would be 3188.12 Hz, which is a 118 ppm at 27 MHz [41].

This SH-SAW sensor chip has a mass sensitivity of 1066 ppm at 250 MHz for a 1 nm gold film thickness change, which is about 9 times higher than that of the 27 MHz QCM. Notably, the operating frequency of this SH-SAW is about 9 times higher than that of the 27 MHz QCM. For both QCMs and SH-SAW sensors, the sensitivity should be proportional to the operating frequency. This result suggests that the SH-SAW and QCM with the same frequency will provide similar mass loading sensitivity.

### 3.3. Mass Loading Sensitivity in Liquid

The sensitivity of SH-SAW delay line sensors for mass loading in water has been studied [31,33]. In experiments performed on an SH-SAW chip for antibody-antigen binding, it was clear that the measured phase shift was much larger than the calculated phase shift due to mass change caused by binding [34]. We found that mass loading sensitivity of the SH-SAW was not high and that the main signal changes could be caused by the viscosity changes of the surface. Here, we calculated the velocities and loss of this SH-SAW delay line sensor in water with different gold thicknesses (116.5, 117.5, and 118.5 nm) as shown in Table 2. As shown in the columns of 0% glycerol-water mixture in Table 2, the mass loading sensitivity of this SH-SAW delay line is 964 ppm per 1 nm of gold film thickness change in water at 117.5 nm.

### 3.4. Viscosity Sensitivity in Liquid

To analyze the viscosity sensitivity of this SH-SAW delay line sensor, we used different concentrations of glycerol-water mixtures with well-known material constants. In our experiments, we obtained the velocity and attenuation changes for different concentrations of glycerol-water mixtures (0, 10, 20, and 30 wt%) at a room temperature of 23 °C.

**Figure 4:**
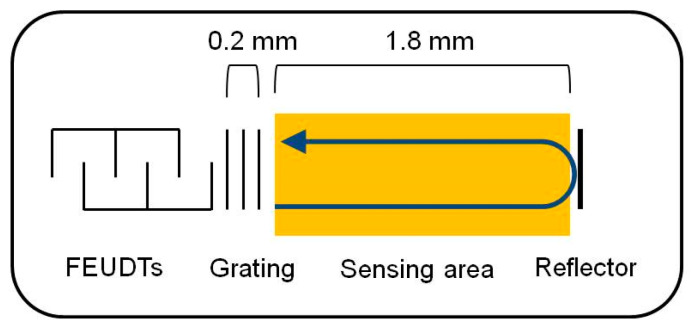
False grating area between the FEUDT and the sensing area.

On the other hand, we calculated the velocity and attenuation of the SH-SAWs in the glycerol-water mixtures using the model shown in Figure 3b. Then, we calculated the phase changes and attenuation changes of the delay line sensor chip in the sensing area using a sensing length of 3.6 mm. The results of the calculations and measurements are shown in Table 3. In the delay line sensor chip, as shown in Figure 4, there are some false grating regions between the FEUDT and the sensing area, which are not included in the sensing length. The total length of these grating areas may be around 0.4 mm, which may cause some errors. In addition, good agreements were obtained between calculations and measurements. However, the calculated results might have some errors due to a measured thickness error of the gold film and/or of the density of the gold film. Although we used the density of the bulk value of the gold, the density of the evaporated gold film might be somewhat smaller than the bulk value. We will be more careful with experiments.

By our calculations, we obtained the viscosity sensitivity of this SH-SAW delay line sensor: 1.10 degrees (16.10 ppm) per 1 wt% change in a 10 wt% glycerol-water mixture. This implies a change of 9.01 degrees (420.14 ppm) per 1 cP (10^−3^ Pa·s) in a 10 wt% glycerol-water mixture. Our calculations and measurements indicate that the mass change caused by antibody-antigen binding on the surface is not sufficient to explain the phase changes in SH-SAW obtained experimentally [34]. In this SH-SAW delay line sensor system, viscosity sensitivity may play a dominant role in antibody-antigen binding events.

## 4. A Simple Method to Fabricate the SH-SAW Biosensor

The SH-SAW POCT platform provides a 3 mm × 5 mm dual-channel SH-SAW biosensor chip as shown in Figure 1; one channel is used for reference and the other for capture. Each channel on the chip has a FEUDT with a center frequency of 250 MHz, a sensing region with an approximately 100 nm thick gold film, and a reflector. The non-substrate material of the chip is a 36Y-cut 90X-propagating quartz with a thickness of 0.5 mm [35,42]. The simple process of biomaterial coating has five steps, including cleaning, cross-linking, biomaterial coating, blocking, and stabilizer coating. The gold sensing area of the chips can be cleaned by physical or chemical methods, such as oxygen plasma cleaning or acetone, which helps to move debris and increase the hydrophilicity of the sensor surface. Oxygen plasma etching at moderate power (<200 W) provides a convenient and efficient method to remove organic agents from gold without causing significant damage [43,44]. The choice of crosslinkers for use on gold sensing surfaces is diverse; dithiobis [succinimidyl propionate] (DSP) is a convenient self-assembling crosslinker that binds to disulfide bonds on gold surfaces within 5 min and provides N-Hydroxysuccinimide (NHS ester) for conjugation of lysine or arginine to the biomaterials.Moreover, other NHS esters containing disulfide bonds, such as 3,3′-dithiobis(sulfosuccinimidyl propionate) (DTSSP), N-succinimidyl 3-(2-pyridyldithio) propionate (SPDP), and N-succinimidyl 6-(3(2-pyridyldithio)propionamido)hexanoate (LCSPDP) can be used to modify the amino groups of antibodies to the gold surface [45,46]. A homogeneous, low cross-reactivity blocking layer is important for biosensor measurements. The choice of blocking agent should be based on the size of the coating materials; blocking agents larger than the coating materials may lead to shielding effects [42]. Sugars, such as sucrose or trehalose, are key materials for the storage and stability of biosensors. Different formulations affect the storage conditions and lifetime of biosensors and are also a key to reach practical application and commercialization [47].

## 5. Quick Whole Blood Measurement by SH-SAW

**Figure 5:**
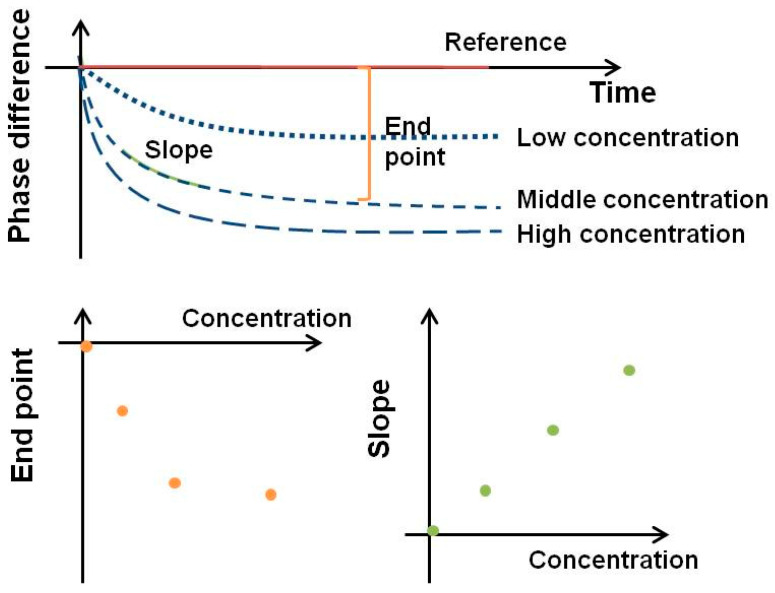
Signal analysis from SH-SAW.

Many in vitro diagnostics (IVD) devices use serum or plasma for clinical measurements. However, serum or plasma samples require pre-separation of blood cells from whole blood using a centrifuge and therefore are not readily available from the laboratory. There is an urgent need for an IVD device that can measure whole blood and can be used at home, in rural areas, or in places where medical resources are scarce [48,49]. Unlike spectroscopic measurements, the detection of the SH-SAW biosensor is not influenced by the transmittance or optical complex reflection factors of the sample. As shown in Figure 5, the SH-SAW biosensor can allow quantitative measurements by measuring real-time immunokinetic binding.

To compare changes in the SH-SAW, the phase shift is analyzed using an endpoint window, which is the signal difference from the phase shift at the initial baseline point to the phase shift at a specific time point. However, endpoint data are more susceptible to spurious initial points and are usually saturated at high concentrations. Another way to analyze real-time data is to use the slope of the real-time curve for a specific range. The advantage of a slope window is that we can quickly get a result in a few seconds. By using a slope window of 20–40 s, a measurement such as a SAW biosensor with an S-protein coating can be completed in less than 40 s. By creating a standard curve using the slope of a specific range of real-time curves, a fast test can be completed in less than a minute [30,50].

During a 5-microliter whole blood measurement, the viscosity and/or concentration of the whole blood sample may change due to evaporation and/or coagulation, and then the propagation characteristics of the SH-SAW may be affected. The shorter the measurement time, the better, but the measurement should be completed within three minutes at the most.

## 6. Detection Range of the SH-SAW Biosensor

As shown in Table 4, SH-SAW detection for whole blood samples ranges from micrograms per milliliter (μg/mL) to milligrams per milliliter (mg/mL) levels. For different detection ranges, different detection methods should be used. The CRP SAW biosensor requires a secondary antibody to enhance the signal to detect CRP antigen concentrations from 1.9 to 118 μg/mL. The Lp(a) SH-SAW biosensor can directly measure finger whole blood samples without additional enhancers to detect Lp(a) from 83 to 1402 μg/mL. In contrast, the detection range of the SH-SAW biosensor coated with S protein for direct measurement was determined to be 48.3 to 1597.2 BAU/mL with a linear regression of 0.9935. Unlike other markers at low detection concentration levels, such as small molecular markers or cancer markers [29,51], which require secondary antibodies or enhancers or amplification of the measured signal, high concentration levels of measurements require different dilutions to differentiate the signal. In the SH-SAW measurement system, dilution is important for lipid mass spectrometry or other measurements of samples at high concentration levels [30]. The detection range of diluted ApoB is 51 to 2022 μg/mL.

## 7. Detection Limit of the SH-SAW Biosensor

**Figure 6:**
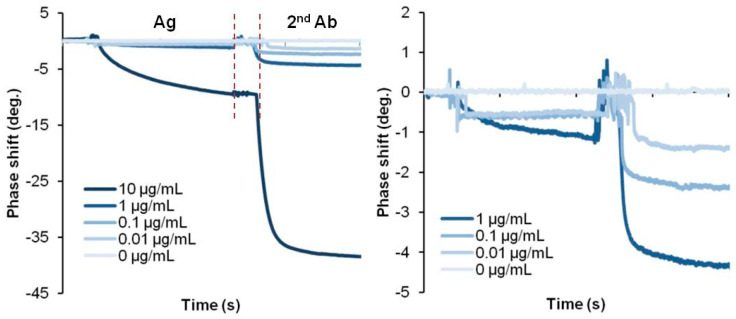
Multiple step measurement of CRP biosensor.

The limit of quantification (LoQ), limit of blank (LoB) and limit of detection (LoD) are parameters to confirm the sensitivity of the biosensor by performing 60 replicate tests at low concentrations to check the standard deviation and coefficient of variation [52]. SH-SAW measurements using secondary antibodies, such as the CRP SAW biosensor, can achieve an LoQ of 1 μg/mL, while direct SH-SAW measurements, such as the Lp(a) SAW biosensor, can achieve an LoD of 83 μg/mL. Direct measurements on the S protein-coated SAW biosensor were determined to have an LoD of 41.91 BAU/mL for the whole blood sample [42]. This SH-SAW biosensor system can directly accept a drop of whole blood on the sensor surface and measure the concentration of the target protein in the sample without any additional washing process. Unlike the SH-SAW biosensor, electrochemical-based measurements or other detection methods can achieve better sensitivity through multiple washing steps or complex processes before and after the measurement, as shown in Table 5. Since whole blood contains red cells, white cells, and many different proteins, unfortunately, these interferences may lead to some errors in the measurements. Nevertheless, this SH-SAW biosensor system can provide results in this harsh environment. The absence of a pretreatment and cleaning system is a clear advantage of this SH-SAW biosensor system and must be highly expected in POCT applications. On the other hand, if we use this SH-SAW biosensor system in a buffer, a very clear signal will be obtained and then the detection limit will be improved. Figure 6 shows some experimental results of CRP detection in a buffer. The figure shows the direct detection method with antigens bound to a capture antibody on the surface and sandwich detection of secondary antibodies binding to a capture antibody bound to the antigens. In this case, the signal at 0.01 μg/mL is easily distinguishable from the signal at 0 μg/mL, and the LoD will be smaller than 0.01 μg/mL. There are also some opportunities to enhance the output signals using gold nano-particles [51,53], horseradish peroxidase (HRP) [54], and gold staining [55]. The detection limit can also be improved by reducing the nonspecific binding. Not only can the blocking agent help prevent nonspecific binding, but some studies have also integrated a transducer into the current biosensor to induce an extra surface shear to remove weakly nonspecifically bound molecules from the sensing surface [56]. Since SAW devices are sensitive to environmental conditions, adjustment or compensation for temperature can also help to improve the sensitivity of the SAW biosensor [57]. In general, acoustic wave-based sensor devices have temperature characteristics that may cause some measurement errors due to the temperature. In the actual testing environment for POCT, attention should be paid to the temperatures of the sensor chips and/or the sample solutions, as they will be outside the laboratory. For this reason, we use a quartz-based SH-SAW sensor, which has a lower temperature drift than LiTaO_3_ or LiNbO_3_-based devices. In addition, in our SH-SAW sensor system, the reference channel helps to compensate for errors due to temperature drifts [58].

## 8. Conclusions

SH-SAW technology has been extensively studied and applied to different applications and quantitative biosensing. The sensitivity to mass loading and viscosity has been well investigated in order to reach the limits and interference of SH-SAW biosensors. The conical cross-linking method between gold sensing surfaces and the biomaterials has been identified for large-scale production. In addition to enabling a fast POCT device, the slope of the real-time signal provides a solution for reducing incubation time. For POCT measurements, there is an urgent need for a whole blood measurement system. The SH-SAW biosensor enables a detection limit of 1 μg/mL for whole blood by premixing enhancers. The SH-SAW devices also have the potential to further improve sensitivity and specificity by premixing biomaterial-conjugated gold nanoparticles, increasing the thickness of the gold film in the sensing area, increasing the detection frequency, or increasing the sensing delay time. The SH-SAW devices show great promise for POCT biosensor development and health management, with the advantages of fast response time, small size, and high sensitivity. As the SAW filters used in cell phones have changed dramatically in the past 20 years, SH-SAW devices for biosensors will change similarly. The size and cost of SH-SAW biosensor devices will reach one-twentieth of their current values in the next 20 years. To achieve this goal, we need to reduce the chip size while improving the device structure. At the same time, multiple markers detection capabilities and improved sensitivity on a chip should be a good strategy. From the commercialization point of view, we are now working on multiple markers detection capabilities on a chip, improving the device structure, and increasing the sensitivity without changing the frequency. On the other hand, from an academic point of view, we are using the simulation program to analyze the bio-layers on the sensor surface.

## Figures and Tables

**Table 1:** Values of liquid layer in the calculations.

	Air	0 wt%	10 wt%	20 wt%	30 wt%	40 wt%
Desity (×10^3^) (kg·m^−3^)	0.0012	0.9967	1.0207	1.0465	1.0714	1.099
Viscosity (×10^−3^) (Pa·s)	0.01845	0.8368	1.22	1.64	2.3	3.4

**Table 2:** The calculated velocities and attenuations of different gold thicknesses and percentage of glycerol.

	Thickness (nm)	Air	Glycerol-Water Mixture				
			0%	10%	20%	30%	40%
Velocity	116.5	4759.59	4754.75	4753.99	4753.02	4751.71	4749.86
(m/s)	117.5	4754.52	4750.17	4749.40	4748.43	4747.11	4745.25
	118.5	4749.43	4744.54	4743.77	4742.78	4741.46	4739.59
Attenuation (dB/λ)	116.5	0.000270	0.0558	0.0647	0.0760	0.0913	0.1131
	117.5	0.000272	0.0562	0.0651	0.0764	0.0918	0.1137
	118.5	0.000274	0.0565	0.0655	0.0770	0.0925	0.1145

**Table 3:** Calculated and measured velocity changes and attenuation changes of different percentages of glycerol. (Normalized at 0% glycerol-water mixtures).

	Glycerol-Water %	10%	20%	30%
	≒Viscosity (×10^−3^ Pa·s)	1.22	1.64	2.30
Phase Shift (degree)	Calculation	−10.99	−25.01	−43.95
	Measurement	−10.43	−23.45	−39.79
Amplitude shift (dB)	Calculation	−1.69	−3.85	−6.77
	Measurement	−1.54	−3.53	−6.13

**Table 4:** Performance of whole blood measurement by SH-SAW biosensor.

Test Item	Marker			
	CRP	Lp(a)	apoB	SARS-CoV-2 S Protein
Detection Method	Pre-mix with secondary antibody	Directly measurement	Dilution measurement	Directly measurement
Test time	3 min	3 min	30 s	40 s
Linearity range	1.9–118 μg/mL	83–1402 μg/mL	51–2022 μg/mL	50–1500 BAU/mL
Sensitivity	LoQ = 1000 ng/mL LoD = 390 ng/mL LoB = 170 ng/mL	LoD = 58.3 g/mL LoB = 14.5 g/mL	LoD = 80.1 g/mL LoB = 54.7 g/mL	LoD = 41.91 BAU/mL LoB = 27.48 BAU/mL
Comparison	R = 0.986/ Slope = 1.003 (n = 170) *	R = 0.9698/ Slope = 1.0598 (n = 55) ^#^	R = 0.9287/ Slope = 0.9741 (n = 55) ^%^	NPA = 98.7% (n = 79)/PPA = 94.3% (n = 35) ^@^

* Compared with Beckman Coulter CRP Latex; ^#^ Compared with Abbot Lp(a) Reagent Kit; ^%^ Compared with Advia Chemistry—Apolipoprotein B; ^@^ Compared with cPass™ SARS-CoV-2 Neutralization Antibody Detection Kit; Positive percentage agreement (PPA); Negative percentage agreement (NPA); Limit of quantification (LoQ); limit of blank (LoB); and limit of detection (LoD).

**Table 5:** Comparison of CRP detection methods based on different technologies.

Detection Method	Sample Type	Sample Size	Test Time	Detection Probe	Detection Range	Limit of Detection	Ref.
Electrochemistry (DPV)	CRP antigen	-	60 min	Antibody	0.01–1000 pg/mL	5.0 pg/mL	[59]
Electrochemistry (SWV)	Serum	-	90 min	Aptamer	1–125 ng/mL	0.0017 ng/mL	[60]
SPR	Serum	-	15 min	Antibody	0.006–70 μg/mL	0.009 μg/mL	[61]
Colorimetric immunoassay	CRP antigen	30 μL	5 min	Aptamer	0.889–20.7 μg/mL	1.2 μg/mL	[62]
Colorimetric immunoassay	CRP antigen	100 μL	30 min (pre-process)	Antibody	10–5000 ng/mL	100 ng/mL	[63]
SAW (Rayleigh wave)	CRP antigen	50 μL	10 min	Antibody	0.1–1000 μg/mL	0.1 μg/mL	[64]
QCM	Serum	-	-	Antibody	0.01–1000 ng/mL	-	[65]
SH-SAW	Finger blood	5 μL	3 min	Antibody	2–120 μg/mL	0.39 μg/mL	This paper
	Serum				-	<10 ng/mL	

Differential pulse voltammetry (DPV); Square wave voltammetry (SWV); Surface plasmon resonance (SPR).

## Data Availability

Not applicable.
